# Extracellular enzyme stoichiometry reveals carbon and nitrogen limitations closely linked to bacterial communities in China’s largest saline lake

**DOI:** 10.3389/fmicb.2022.1002542

**Published:** 2022-09-21

**Authors:** Weizhen Zhang, Yongqin Liu, Mengdie Geng, Ruirui Chen, Jiyi Wang, Bin Xue, Ping Xie, Jianjun Wang

**Affiliations:** ^1^Center for The Pan-Third Pole Environment, Lanzhou University, Lanzhou, China; ^2^State Key Laboratory of Lake Science and Environment, Nanjing Institute of Geography and Limnology, Chinese Academy of Sciences, Nanjing, China; ^3^State Key Laboratory of Soil and Sustainable Agriculture, Institute of Soil Science, Chinese Academy of Sciences, Nanjing, China; ^4^College of Life Science and Technology, Harbin Normal University, Harbin, China; ^5^State Key Laboratory of Plateau Ecology and Agriculture, College of Eco-Environmental Engineering, Qinghai University, Xining, China; ^6^University of Chinese Academy of Sciences, Beijing, China

**Keywords:** extracellular enzyme, microbial metabolic limitations, saline lakes, bacterial community composition, sediment carbon storage

## Abstract

Saline lakes possess substantial carbon storage and play essential roles in global carbon cycling. Benthic microorganisms mine and decompose sediment organic matter via extracellular enzymes to acquire limiting nutrients and thus meet their element budgets, which ultimately causes variations in sediment carbon storage. However, current knowledge about microbial nutrient limitation and the associated organic carbon changes especially in saline lake remains elusive. Therefore, we took Qinghai Lake, the largest saline lake of China, as an example to identify the patterns and drivers of microbial metabolic limitations quantified by the vector analyses of extracellular enzyme stoichiometry. Benthic microorganisms were dominantly colimited by carbon (C) and nitrogen (N). Such microbial C limitation was aggravated upon the increases in water salinity and sediment total phosphorus, which suggests that sediment C loss would be elevated when the lake water is concentrated (increasing salinity) and phosphorus becomes enriched under climate change and nutrient pollution, respectively. Microbial N limitation was predominantly intensified by water total nitrogen and inhibited by C limitation. Among the microbial drivers of extracellular enzyme investments, bacterial community structure consistently exerted significant effects on the C, N, and P cycles and microbial C and N limitations, while fungi only altered the P cycle through species richness. These findings advance our knowledge of microbial metabolic limitation in saline lakes, which will provide insights towards a better understanding of global sediment C storage dynamics under climate warming and intensified human activity.

## HIGHLIGHTS

- C and P cycles are regulated by sediment pH, while N cycle by water TN.

- Increasing salinity and sediment phosphorus aggravate microbial C limitation.

- Microbial N limitation increases with rising water TN and reducing C limitation.

- Bacterial beta-diversity drives C, N and P cycles and microbial nutrient limitation.

## Introduction

Saline lakes, which account for approximately 50% of total inland waters (Wetzel, 2001), tend to be essential components for carbon storage and play important roles in global carbon cycling (Curtis and Adams, 1995; Waiser and Robarts, 1995; Song et al., 2018b; Wen et al., 2018; Chen et al., 2021). Saline lakes contain higher concentrations of dissolved organic carbon (DOC) than fresh waters due to the evaporative condensation effect (Curtis and Adams, 1995; Song et al., 2017). Ninety-two percent of the lakes on the Tibetan Plateau lakes are saline and located in areas with prolonged sunshine and arid conditions (Zhang et al., 2011), and they possess 13.39 Tg C, which is approximately 84.3% of the DOC storage in lakes across China (Song et al., 2018a). Moreover, the Tibetan Plateau has the largest inland saline lake in China: Qinghai Lake, which presents a higher level of buried surficial sediment organic carbon than many other large freshwater lakes in China, such as Taihu Lake (Chen et al., 2021). Heterotrophic microorganisms mineralize organic matter to create the trophic base for detrital food webs (Benstead et al., 2021), forming the microbial loop that accounts for a crucial portion of the energy and nutrient flow in most aquatic ecosystems, thus driving global carbon and nutrient cycles (Cherabier and Ferriere, 2022; Villalba et al., 2022). Extracellular enzymes are secreted by the microbial assemblage to participate in the decomposition of organic sources and subsequent acquisition of limiting nutrients (Hill et al., 2006). Typically, microbial activity and extracellular enzyme activity (EEA) are the highest in the top surficial layer of sediments (Arnosti, 2011). Nutrient limitation may induce microbial mining for resources from persistent organic matter in deeper layers, which may influence ecosystem carbon storage (Hicks et al., 2021). However, current knowledge about microbial nutrient limitation and associated organic carbon dynamics in saline lake sediments remains elusive.

The degradation of organic matter by extracellular enzymes is a rate-limiting process for the detrital food webs in transferring energy and nutrients to the heterotrophic microorganisms from autotrophic producers (Nannipieri et al., 2002). In lake sediments, microorganisms often face an imbalance in resources availability and thus suffer from nutrient limitation (Hill et al., 2018). To adapt to resource constraints, microbes regulate their relative allocation of EEA directed toward the acquisition of C, N, and P to meet their elemental budgets (Sinsabaugh and Moorhead, 1994; Penton and Newman, 2008). Ecoenzymatic stoichiometry is defined as the relative activity of extracellular enzymes involved in C, N, and P cycling, and it is primarily applied to indicate the microbial nutrient limitation (Sinsabaugh et al., 2009; Moorhead et al., 2016). Sinsabaugh et al. (2008, 2009) and Sinsabaugh and Shah (2012) revealed that the activities of key enzymes that catalyzing the hydrolysis of principal C, N, and P compounds are characterized by similar scaling relationships, with a mean ratio for C:N:P extracellular enzyme activities near 1:1:1 in soils, biofilms and sediments. Based on these ecoenzymatic stoichiometry ratios, Moorhead et al. (2013, 2016) proposed a method of vector analysis to quantify the relative investments in C *vs* nutrient acquisition and P *vs* N acquisition by calculating the length and angle of vectors in plots of C:N vs. C:P enzymes activities, respectively (). The vector lengths and angles reflect the relative resource limitations co-existing in microbial communities, and their values are independent of variations in total enzyme activity (Moorhead et al., 2013, 2016).

The microbial resource limitation deduced from the ecoenzymatic stoichiometry ratio is sensitive to environmental variations (Wang et al., 2020). For example, in drylands with aridity >0.70, the enzymatic C:nutrient (N and P) ratios decline significantly as the aridity increases, and the enzymatic N:P ratios are generally higher than those with aridity <0.70 (Feng et al., 2019). The elevated litter N:P increases P limitation in heterotrophic microbes associated with submerged plant litter, and thus leads to higher relative activity of P-acquiring enzymes and lower enzymatic N:P ratio (Francoeur et al., 2020). Furthermore, the microbial nutrient limitation may have the potential to predict changes in ecosystem C storage, which are mainly studied in soil ecosystems (Wang et al., 2020; Cui et al., 2021). For example, higher soil temperature and moisture in lower elevations decrease the microbial C limitations in the rhizosphere, which suggesting that global warming may decrease the microbial relative investments in producing enzymes involved in C decomposition and thus be conducive to the retention of soil organic C (Cui et al., 2021). On the contrary, N/P addition significantly aggravates microbial C limitation while alleviates N/P limitation, which may increase the investments in acquiring C and thus induce soil C loss (Luo et al., 2017; Chen et al., 2018; Zheng et al., 2020). Analogously, it’s proved that heavy metal stress aggravates microbial C limitation and may potentially promote soil C loss (Wang et al., 2020). These results also emphasize the significance of research on the ecoenzymatic stoichiometry and its potential role in the variations in sediment organic carbon in saline lakes; However, relevant studies have not yet been reported.

Benthic microbial communities are considered as key players in sediment biogeochemical cycling through the absorption, consumption and transformation of resources (i.e., C, N, and P) (Battin et al., 2016; Wang et al., 2022). Generally, nutrient limitation drives the spatial organization of microbial groups and therefore determines the distribution of extracellular enzymes released by microbes for nutrient acquisition (Mitri et al., 2016). Microbial diversity may also affect the enzyme pool composition and then the potential for synergistic interactions among extracellular enzymes (Sinsabaugh, 2005). Notably, resource acquisition by different microbial taxonomic groups, such as bacterial and fungal communities, proceeds by distinct pathways (LaRowe et al., 2020; Hicks et al., 2021). Bacteria possess higher metabolic diversity than fungi because of the tremendously diverse phylogenetic subgroups that contain genes encoding secreted enzymes capable of degrading organic matter, such as proteins and carbohydrates (Orsi, 2018; LaRowe et al., 2020). For fungi, their hyphal organization is capable of assimilating nutrients along a distributed network and focusing the release of the extracellular enzyme at the growing tips (Frey et al., 2003); thus, they are more efficient at colonizing and cleaving large detrital particles than bacteria (Sinsabaugh, 2005). Whether bacterial or fungal communities dominate the response of microorganisms to resource constraints has not been clarified, and the potential explanations produce inconsistent results in different situations (Carrara et al., 2018; Benito-Carnero et al., 2021). For example, strong nutrient limitations and low-quality carbon favor fungal over bacterial decomposers, which suggests a dominant functional role of the fungal community in litter decomposition (Benito-Carnero et al., 2021). In contrast, N fertilization may reduce belowground C allocation from plants, thus leading to a shift in bacterial but not fungal community composition and the accompanying declines in EEA (Carrara et al., 2018). Therefore, disentangling the relative contributions of bacterial and fungal communities to sediment resource acquisition is an indispensable step toward revealing the mechanism underlying biogeochemical cycling in saline lake sediment.

Qinghai Lake is located in the northeast of the Tibetan Plateau, which has faced evident climate changes in recent decades, such as increased temperature and fluctuating precipitation (Fan et al., 2021). In recent years, Qinghai Lake has experienced significant environmental issue such as nutrient enrichments of nitrogen and phosphorus resulting from the rapid development of travel, industrial and agricultural activities (Ao et al., 2014; Chen et al., 2021). In the context of climate warming and intensified human activity, dissecting the mechanisms underlying microbial nutrient limitation and the potential influences of such limitation on C storage in saline Qinghai Lake will provide insights toward a better understanding of global C cycling. Accordingly, we investigated the microbial groups, including bacterial and fungal communities, and EEAs involved in C, N and P cycling based on the surface sediments covering Qinghai Lake. We aim to answer the following questions for saline lakes, such as Qinghai Lake: (1) What are the patterns of microbial metabolic limitation? (2) What are the abiotic and biotic drivers of microbial metabolic limitation and the resultant potential impacts on lake sediment C storage?

## Materials and methods

### Study site and sampling

Qinghai Lake (36°32′–37°15′N, 99°36′–100°16′ E) is the largest saline lake in China, with an area of approximately 4,472 km^2^ and an average water depth of 25 m. Located at the intersection of the eastern Asian monsoon, the northwestern arid region and the northeastern Tibetan Plateau, Qinghai Lake has high climatic sensitivity with dry, cold and windy conditions, strong solar radiation and a large diurnal temperature change of –10°C (Dong et al., 2019). In September 2020, 23 surface sediment samples (top of 5 cm) covering the whole Qinghai Lake were retrieved with a box sampler (Supplementary Figure 1). Each surface sediment sample was separated into two subsamples that were stored at –20 and 4°C until analysis. The former was used for the microbial community and EEA analyses and the latter was used for chemical measurements. Freezing is a procedure recommended for sample preservation when the EEA cannot be determined immediately (Hewins et al., 2016). For the corresponding water chemical parameters at each site, 1 L of overlaying water from the lake surface layer (upper 50 cm) was collected by a 5-L Schindler sampler. For each sampling site, in situ measurements of environmental variables were also conducted including the water depth and Secchi depth (SD) (using a bathymeter and a Secchi disk, respectively), water temperature, dissolved oxygen concentration, salinity, pH and conductivity in the water column (using a multiparameter water quality detector, YSI Incorporated, Yellow Springs, OH, USA). The pH and conductivity in the surface sediment were measured by a pH and a conductivity meter.

### Analysis of chemical variables

The surface sediment samples were freeze-dried to constant weights, ground into a fine powder, and then passed through a 100-mesh sieve for total carbon (TC), total nitrogen (TN), and total phosphorus (TP) analyses. The sediment TC and TN were measured by an elemental analyzer (Flash EA 1112 series, CE instruments, Italy). The sediment TP was determined by molybdenum blue colorimetry after a digesting procedure using hydrofluoric acid (HF)-perchloric acid (HClO_4_) (Sparks, 1996). TN and TP in water were measured following the standard method (Huang et al., 2000).

### Bacterial and fungal communities

The sediment genomic DNA was extracted using the DNeasy PowerSoil Kit (QIAGEN, Germany) following the manufacturer’s protocols. For bacteria, we chose the universal primers [515F, 5′-GTGYCAGCMGCCGCGGTAA-3′ and 806R, 5′-GGACTACNVGGGTWTCTAAT-3′] to amplify the V4 region of the 16S ribosomal RNA gene using the polymerase chain reaction (PCR) in triplicate and then mixed the replicates. We obtained the barcoded PCR products and normalized them at equal molar concentration, and then sequenced the products on the Illumina HiSeq sequencing platform (Illumina Inc.) with a 2 × 250 bp paired-end. For fungi, we chose the universal primers [gITS7F, 5′-GTGARTCATCGARTCTTTG-3′ and ITS4R, 5′-TCCTCCGCTTATTGATATGC-3′] to amplify the internal transcribed spacer 2 (ITS2) region of the nuclear ribosome. We pooled the purified amplicons in equimolar amounts and sequenced the products in the same HiSeq run. The details for bioinformatic analyses and sequence processing of bacteria and fungi are described in a previous study (Zhang et al., 2021a).

### Assays of extracellular enzyme activities

Five key extracellular enzymes involved in catalyzing the terminal reactions that hydrolyze assimilable products from the principal C, N and P sources were measured by a fluorimetric microplate enzyme assay (Pritsch et al., 2004) according to a published protocol (Liu et al., 2021). β-1,4-Glucosidase (BG) and cellobiohydrolase (CBH) are related to C cycling for cellulose degradation, β-1,4-N-acetylglucosaminidase (NAG) and leucine aminopeptidase (LAP) are related to N cycling for chitin and polypeptide degradation, respectively; and alkaline phosphatase (AP) is related to P cycling for phospholipid and phosphosaccharide degradation. The EEA was finally expressed as nanomoles of substrate released per hour per gram of dry sediment (nmol g sediment^–1^ h^–1^). So far, the sediment studies on EEAs and ecoenzymatic stoichiometry are mainly focused on freshwater ecosystems (Sinsabaugh et al., 2009, 2012; Xiao et al., 2018; Kohler et al., 2020), while lack in saline lakes especially lakes in Qinghai-Tibet Plateau. Thus, to recognize the status and characteristics of EEAs and ecoenzymatic stoichiometry of Qinghai Lake sediment, we compared them with those in typical freshwater lakes across China, including Fuxian Lake in the southwestern Yunnan-Guizhou Plateau, 38 shallow lakes in the Yangtze–Huaihe River basin (unpublished data) and Hulun Lake in the northern semiarid area (Zhang et al., 2021a).

### Calculation of microbial metabolic limitation

Stoichiometric approaches based on nutrient chemistry and extracellular enzymes were used to evaluate the relative nutrient limitation in water and sediment, respectively. For water, the well-known Redfield ratio of 16N: 1P was applied (Redfield, 1958). For sediment, a vector analysis based on ecoenzymatic stoichiometry for both the determination and quantification of metabolism limitation (Moorhead et al., 2016) was performed, and it reflects the relative resource demands of the microbial community independent of the variations in total EEAs. The vector analysis of ecoenzymatic stoichiometry was conducted according to the following equations (Moorhead et al., 2016).


(1) $$C\,\mathrm{limitation}=\mathrm{Vector}\,\mathrm{length}=x^{2}+y^{2}$$

(2) $$\mathrm{Vector}\;\mathrm{angle}\;\left({}^{\circ }\right)=\mathrm{DEGREES}\;\left(\mathrm{ATAN2}\;\left(\text{x},\text{y}\right)\right)$$

(3) $$N\;\mathrm{limitation}=90^{\circ }-\mathrm{vector}\;\mathrm{angle}$$

where x is the relative activity of C versus P-acquiring enzymes; y is the relative activity of C versus N-acquiring enzymes; Vector length is the square root of the sum of x^2^ and y^2^, which is used to quantify microbial C limitation (Eq. 1); and Vector angle is the arctangent of the line extending from the plot origin to point (x, y), which is used to quantify microbial N or P limitation (Eq. 2). Higher vector length values indicate higher microbial C limitation. Vector angles >45° and <45° represent microbial P limitation and N limitation, respectively. Microbial P limitation is positive to the vector angle, while N limitation is negative to it. For a better understanding, we defined 90° minus the vector angle as the value of N limitation, which is positive to the intensity of microbial N limitation (Eq. 3).

### Statistical analysis

The C, N, and P cycles that characterize nutrient acquisition efforts through enzymatic activities, were calculated by summing enzyme activities involved in acquiring C (AG+BG), N (NAG+LAP) and P (AP), respectively. Pearson correlation analysis was performed to explore the relationships of the five enzyme activities and microbial metabolic limitations with abiotic variables. Ordinary least squares (OLS) regression was adopted to confirm the relationships between microbial C limitation and microbial N limitation as well as the correlations of microbial metabolic limitations or the C/N/P cycle with environmental variables and microbial species richness. Linear models were applied to visualize the relationships underlying the differences in microbial metabolic limitation, the C/N/P cycle or the element-cycle with the Bray-Curtis dissimilarity of the bacterial and fungal community compositions, and then the Mantel test (999 permutations) was performed to evaluate the significance of these linear models. The element cycle is a matrix that includes the activity of five enzymes involved in the C, N and P cycles, and the difference in the element cycle was calculated by the Euclidean distance between each pair of sampling sites. We did not use the sum value of the five enzyme activities to represent the element cycle due to the large differences among the values of EEAs involved in the C, N and P cycles which might underestimate the weight of variations in enzymes with lower activities.

Random forest (RF) analysis was used to quantify the relative contributions of environmental factors to microbial species richness, microbial metabolic limitation and the C/N/P cycle. Redundancy analysis (RDA) was used to determine the abiotic factors with a significant effect on the bacterial and fungal community structures. Finally, the structural equation model (SEM), which aims to quantify the potential causal relationships among variables (Grace et al., 2012), was simulated to clarify the relationships among abiotic factors, bacterial and fungal community attributes including species richness and community compositions, the C/N/P cycle and microbial metabolic limitations. In the SEM, the abiotic factors were selected based on the multiple stepwise regressions of the microbial C and N/P limitations. We Z score-transformed the input variables to allow cross-comparisons and performed an analysis of variance (ANOVA) to estimate the significance of the standardized path coefficient (β) and the model. The final selected model met the criteria as previously mentioned (Zhang et al., 2021b). The standardized total effect (SE) of each variable on microbial C or N limitation was then calculated by the significant path coefficient, which included both direct and indirect correlations.

## Results

### Physiochemical properties, microbial community compositions, EEAs, and the patterns of microbial metabolic limitation

A low nutrient level with a mean water TP content of 0.04 mg L^–1^ was observed across the Qinghai Lake (Supplementary Table 1), and the mean value of the water TN:TP ratio was above the Redfield ratio of 16N: 1P, indicating P limitation. For the surface sediments of Qinghai Lake, the mean values of TC, TP and TN contents were 65.25, 0.62, and 2.89 g kg^–1^, respectively (Supplementary Table 1), and the mean value of the sediment TN:TP ratio was 10.19.

A total of 78 phyla and 337 families of bacteria, and 8 phyla and 144 families of fungi were determined across the sediments of Qinghai Lake. For bacteria, Proteobacteria, Bacteroidetes and Chloroflexi were the top phyla with the mean relative abundances of 37.48, 15.01, and 13.88%, respectively, followed by Firmicutes (6.57%), Actinobacteria (3.88%), Planctomycetes (3.80%), and Chlorobi (3.26%) (Supplementary Figure 2A). For fungi, the most dominant phyla identified were Ascomycota (33.32%) and Basidiomycota (15.79%), followed by Rozellomycota (1.57%) and Chytridiomycota (1.03%) (Supplementary Figure 2B).

For EEA alone, BG and NAG (458.12 and 114.38 nmol MUF g^–1^ h^–1^, respectively) were respectively the primary decomposers of C- and N-containing substrates, which showed absolute advantages over CBH (159.11 nmol MUF⋅g^–1^ h^–1^) and LAP (24.9 nmol AMC⋅g^–1^ h^–1^), respectively (Supplementary Table 1).

**FIGURE 1:**
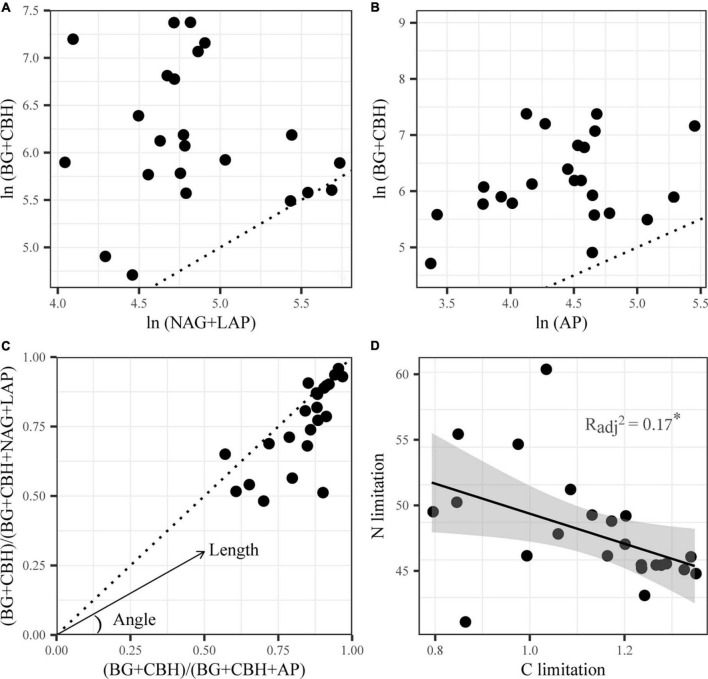
Stoichiometry of the relative proportion of C- versus N-acquiring enzymes **(A)**, C- versus P-acquiring enzymes **(B)** and enzymatic C:N versus C:P **(C)**, and linear correlations between microbial C and N limitation **(D)** of Qinghai Lake sediments. BG, β-1,4-glucosidase; CBH, β-D-cellobiosidase; NAG, β-1,4-N-acetylglucosaminidase; LAP, L-leucine aminopeptidase; AP, alkaline phosphatase. A 1:1 dotted line is superimposed **(A–C)**, which indicates equal acquisition effort by the compared element-acquiring EEA on the x and y axis (Sinsabaugh et al., 2009). Deviations from the 1:1 line imply limitation in favor of the enzyme group where more acquisition effort is directed.

Colimitations of carbon and nitrogen characterized the sediment microorganisms of Qinghai Lake according to the vector analysis of enzymatic stoichiometry. On the one hand, EEAs involved in C acquisition were consistently higher than those involved in N and P acquisitions (Figures 1A,B), which implied a greater C constrain than N and P constraints. On the other hand, almost all data points were below the diagonal line and had vector angles <45° (Figure 1C), which indicated microbial N limitation rather than P limitation. Additionally, significant negative correlations between C limitation and N limitation were identified by linear regression (*p* < 0.05, Figure 1D).

### Effects of single physiochemical properties and microbial community attributes on microbial metabolic limitation

**FIGURE 2:**
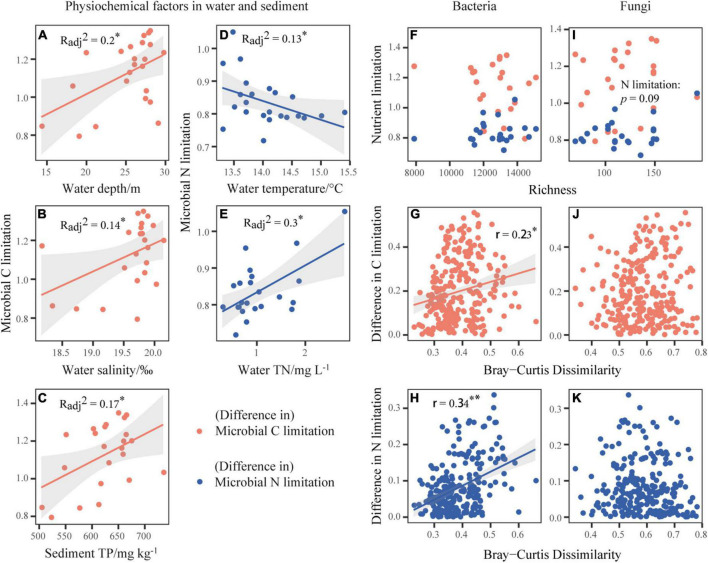
Correlations of the microbial metabolic limitations with physiochemical factors in water and sediment **(A–E)** or bacterial and fungal community richness **(F,I)**. And the relationships between bacterial **(G,H)** or fungal **(J,K)** community composition represented by Bray-Curtis dissimilarity and difference in microbial metabolic limitations. The orange dot indicates the vertical axis variable as microbial C limitation or difference in microbial C limitation. The blue dot indicates the vertical axis variable as microbial N limitation or difference in microbial N limitation. The lines represent the fitted linear regressions with 95% confidence intervals indicated by the shaded areas, where only significant relationships are shown. The adjusted *R*^2^ or *r* values of the linear models are denoted. *p < 0.05, **p < 0.01.

Microbial C limitation increased significantly with increases in water salinity, sediment TP and water depth (*p* < 0.05, Figures 2A–C and Supplementary Figure 3). Microbial N limitation decreased and increased significantly with increasing water temperature and water TN, respectively (*p* < 0.05, Figures 2D,E and Supplementary Figure 3). For the enzymes involved in C cycle, CBH was positively related with water temperature (*p* < 0.05, Supplementary Figure 3). For those involved in N cycle, NAG and LAP were both promoted by water TN (*p* < 0.05, Supplementary Figure 3), NAG was negatively related to the water depth and SD, while LAP was positively associated with sediment TC. For the enzyme involved in P cycle, AP was negatively related to sediment TP (*p* < 0.05, Supplementary Figure 3). Under the slight fluctuation of pH values from 9.40 to 10.25 across Qinghai Lake, the activities of BG, CBH, NAG, and AP changed in the hump-shaped patterns according to the quadratic regressions (*p* < 0.05, Supplementary Figures 4B,D,G).

Among the microbial community attributes, including the richness (Figures 2F,I) and community structures (Figures 2G,H,J,K) of bacteria and fungi, we found the consistent influences of bacterial community structure on element cycling and microbial metabolic limitations. Specifically, increases in the bacterial Bray-Curtis dissimilarity significantly increased the differences in C, N, P, and element cycles (*p* < 0.05, Supplementary Figures 6A–C,G) and the differences in microbial C and N limitations (*p* < 0.05, Figures 2G,H). For fungi, however, it only inhibited the P cycle via the variation in richness (*p* < 0.01, Supplementary Figure 7).

### Relative contributions of factors that driving the variations in microbial community attributes, the C/N/P cycle and microbial metabolic limitation

**FIGURE 3:**
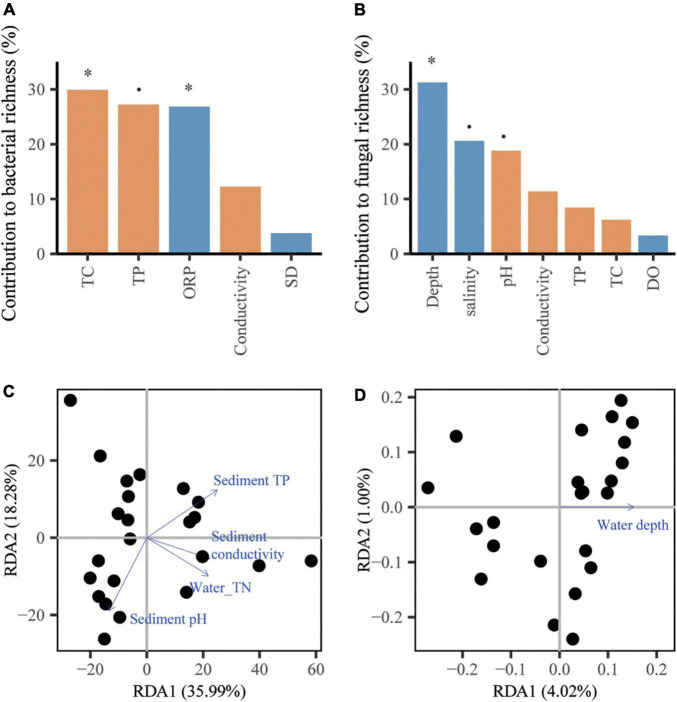
The contributions of abiotic factors on microbial community attributes. The panels include random forest analyses of the richness of bacteria **(A)** and fungi **(B)**, and redundancy analysis (RDA) of the community compositions of bacteria **(C)** and fungi **(D)** represented by biplot graph. The columns filled with yellow and blue indicate sediment and water variable, respectively. The significance of each variable was shown above the column. *p* < 0.1, *p < 0.05.

For bacteria, the richness was influenced by the sediment TC and water ORP (*p* < 0.05, Figure 3A) and the community structure was influenced by water TN and sediment variables, including conductivity, pH and TP (*p* < 0.05, Figure 3C). For fungi, water depth accounted for 31.23% of the variations in richness (*p* < 0.05, Figure 3B), while abiotic factors showed a weak influence on the community structure with a low total explanation of 5.02% from the first and second axes of the RDA (Figure 3D).

**FIGURE 4:**
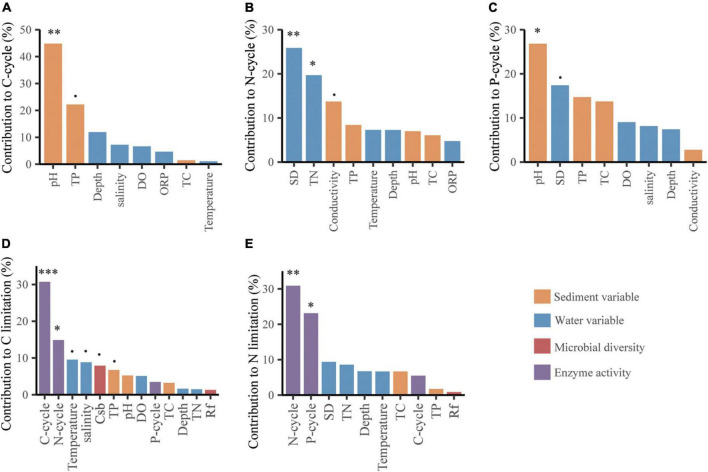
The contributions of abiotic factors on C-/N-/P-cycle **(A–C)**, and of abiotic and biotic factors on microbial C **(D)** and N **(E)** limitations based on random forest analyses. The columns filled with yellow, blue, red and violet indicate sediment variable, water variable, microbial diversity and enzyme activity, respectively. The microbial diversity includes the richness of bacteria (Rb) and fungi (Rf), and the community structure of bacteria (CSb) and fungi (CSf) represented by the first axis of detrended correspondence analysis of the communities. The significance of each variable was shown above the column. *p* < 0.1, *p < 0.05, **p < 0.01, ***p < 0.001.

Microbial C limitation was mainly altered by the C and N cycles with relative contributions of 30.73 and 14.87%, respectively (*p* < 0.05, Figure 4D), followed by water temperature, salinity, bacterial community structure and sediment TP with relative contributions of 9.53, 8.83, 7.89, and 6.75%, respectively (*p* < 0.1, Figure 4D). Microbial N limitation was primarily changed by the N and P cycles with relative contributions of 30.88 and 23.12%, respectively (*p* < 0.05, Figure 4E). Sediment pH dominated the variations in the BG and CBH enzymes and C and P cycles with relative contributions of 41.48, 31.78, 44.83, and 26.81%, respectively (*p* < 0.05, Figures 4A,C and Supplementary Figures 8A,B). In addition, the water SD and TN dominated variations in the N cycle with relative contributions of 25.85 and 19.69%, respectively (*p* < 0.05, Figure 4B).

### Direct and indirect relationships of physiochemical properties, microbial community attributions and the C/N/P cycle with microbial metabolic limitation

**FIGURE 5:**
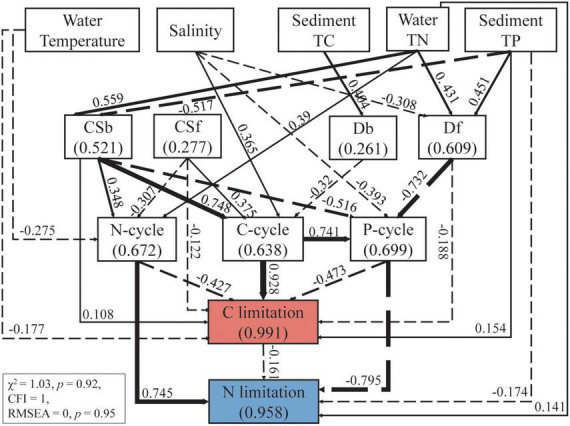
Structural equation model (SEM) showed the effects of main environmental factors and microbial community attributes on sediment microbial C and N limitation. The environmental factors were selected according to stepwise multiple linear regressions between measured physiochemical factors and carbon limitation or nitrogen limitation (Supplementary Table 3). The microbial community attributes include the richness of bacteria (Rb) and fungi (Rf), and the community structure of bacteria (CSb) and fungi (CSf) represented by the first axis of detrended correspondence analysis of the communities. Continuous and dashed arrows denote positive and negative relationships respectively at the significance level of *p* < 0.05. Numbers adjacent to the arrows are standardized path coefficients (β) indicating the effect sizes, and the arrow width is proportional to β.

**FIGURE 6:**
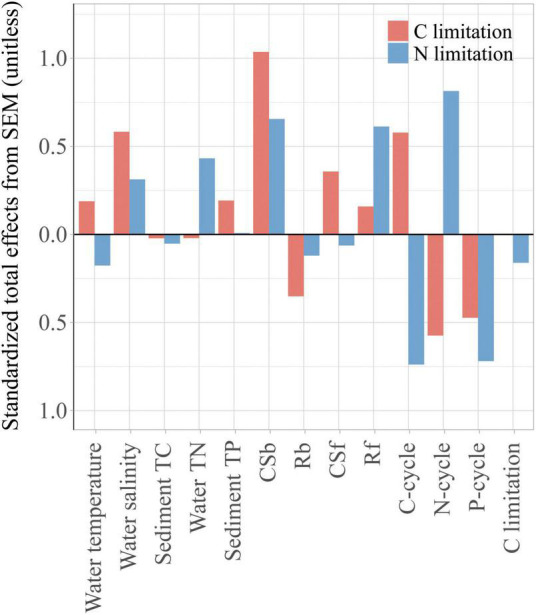
The standardized total effects of each explanatory variable for C and N limitation based on SEM. The microbial community attributes include the richness of bacteria (Rb) and fungi (Rf), and the community structure of bacteria (CSb) and fungi (CSf) represented by the first axis of detrended correspondence analysis of the communities.

The multiple stepwise regressions screened water temperature, salinity, sediment TC and sediment TP as the main factors responsible for the variation in microbial C limitation, with a total contribution of 49.8%. Water temperature and water TN were the dominant variables, which together explained 40.2% of the variations in microbial N limitation (*p* < 0.01, Supplementary Table 3). With the inclusion of these selected physiochemical properties, microbial community attributes, and C, N, and P cycles, the final SEM explained 99.1 and 95.8% of the variations in microbial C and N limitations, respectively (Figure 5). The results show that microbial community attributes contributed more to both C and N limitations compared with physiochemical properties (Figure 6).

For C limitation, water salinity was the largest physiochemical contributing factor (SE = 0.583), followed by sediment TP and water temperature (Figure 6, SE = 0.192 and 0.188, respectively). Water salinity indirectly affected C limitation via C and P cycles and fungal richness (Rf) (Figure 5, β = 0.365, –0.393 and –0.308, respectively). The most important microbial attribute for C limitation was bacterial community structure (Csb) (Figure 6, SE = 1.036), which impacted C limitation directly (β = 0.108) and indirectly via the C, N and P cycles (Figure 5, β = 0.748, 0.348 and –0.516, respectively).

For N limitation, water TN and salinity were the first and second physiochemical contributors (Figure 6, SE = 0.432, 0.312, respectively). N limitation was affected by water TN directly (β = 0.141) and indirectly via the N cycle (β = 0.39), and by salinity indirectly via the P cycle with a hump pattern (β = -0.393, Figure 5) (Supplementary Figure 5). Csb and Rf presented large and similar contributions to N limitation (Figure 6, SE = 0.655 and 0.612, respectively), with the former having an indirect effect via the N and P cycles and the latter via the P cycle (Figure 5).

## Discussion

In this study, we deciphered the patterns of microbial metabolic limitation and their driving mechanisms in Qinghai Lake, the largest saline lake in China. Our results indicated that in the sediments of Qinghai Lake, (1) microorganisms were dominantly limited by C and N; (2) salinity and sediment phosphorus enrichments promoted microbial C limitation; elevated water TN, decreased temperature and reduced C limitation enhanced microbial N limitation; and the bacterial community structure consistently dominated variations in microbial nutrient cycles and C and N limitations.

For the sediments of Qinghai Lake, microbes were primarily C-limited and secondarily nutrient-limited, with as much as 67% of the EEAs directed toward C acquisition (Supplementary Table 2). A higher allocation of EEA directed toward C than nutrient acquisition had also been found in the river and stream sediments (Hill et al., 2012). However, this phenomenon was not found in the sediments of some freshwater lakes, such as Fuxian Lake, which showed almost equal microbial efforts toward C, N, and P acquisitions (Unpublished data). Moreover, the higher mean value of vector length indicated a stronger C limitation in Qinghai Lake than in freshwater lakes, such as Fuxian Lake, Hulun Lake and lakes in the Yangtze–Huaihe River basin (*p* < 0.001, Supplementary Table 2). We found that the mean value of sediment TC content in Qinghai Lake was substantially higher than that in freshwater lakes, including Lake Hulun and Lake Fuxian (*p* < 0.001, Supplementary Table 2). In saline lakes such as Qinghai Lake, the stronger C limitation despite the higher sediment TC contents relative to that in freshwater lakes could be explained by the following two points. On the one hand, previous studies on the relationships between C supply and EEA generally concluded that the absolute enzyme activity, such as glycosidase and peptidase activity, increases with increasing sediment organic matter content (Boschker and Cappenberg, 1998; Shackle et al., 2000; Harbott and Grace, 2005). Compared with freshwater lakes, the higher sediment TC contents in Qinghai Lake might thus determine its higher EEAs. For example, the activities of BG, CBH, NAG, and AP in Qinghai Lake showed dramatically higher values than those in the freshwater lakes listed in Supplementary Table 2 (*p* < 0.001). On the other hand, the EEAs could be influenced by other factors, such as water salinity. At water salinity >19.5‰, the activities of NAG and AP in Qinghai Lake decreased quickly with increasing salinity (Supplementary Figures 5D,G), which increased the gaps between EEAs involved in C and nutrient acquisition and therefore led to strong C limitation in Qinghai Lake.

The sediment N limitation in Qinghai Lake was consistent with previous sediment studies in lakes and coastal wetlands (Hill et al., 2006), gulf hypoxic zones (Hill et al., 2014), streams and rivers (Hill et al., 2012) and lakes such as Hulun Lake (Supplementary Table 2). However, there are other patterns of nutrient limitations found such as in some sediments and wetlands. For example, the sediments of mangroves (Luo and Gu, 2018) and tidal wetlands (Zhai et al., 2022) are characterized by P limitation. The great rivers of the Upper Mississippi River basin (Hill et al., 2009) and the lakes in the Yangtze–Huaihe River basin (Supplementary Table 2) showed shifts in N and P limitations of sediment across sites. Sediment N limitation is generally ascribed to relatively N depletion and P enrichment due to P adsorption to sediments or P recycling, such as in streams and rivers (Hill et al., 2012) and the gulf hypoxic zone (Hill et al., 2014). This might also determine the microbial N limitation in Qinghai Lake given its similar mean sediment TN:TP ratio (10.19) to the N-limited streams (9.88) (Hill et al., 2012).

Increasing sediment phosphorus and salinity aggravated microbial C limitation, which implies intensifying sediment C loss. A similar phenomenon was found in a phosphorus fertilizer addition experiment in forest soil, which showed that phosphorus addition caused microbial C limitation due to the relative increases in activity toward C acquisition with respect to both N- and P-acquiring enzymes (DeForest and Moorhead, 2020). Intriguingly, these findings provided insights toward a better understanding of the potential mechanisms of changes in the carbon storage in saline lakes. For example, although eutrophic lakes have higher total organic carbon burial and accumulation rates (Anderson et al., 2014), a higher organic carbon mineralization rate induced by eutrophication leads to a low long-term burial efficiency of organic carbon (Mendonça et al., 2017; Huang et al., 2019). In this scenario, elevating hydrolysis of organic carbon, i.e., enhanced microbial C limitation, induced by increasing TP might be an additional reason for sediment C loss in saline lakes, such as Qinghai Lake. According to a 42-day laboratory incubation of lake sediments with three salinity gradients, increasing salinity was shown to significantly promote the positive priming effect of sediment, which is defined as an increase in organic carbon mineralization in response to inputs of fresh organic matter (Yang et al., 2020). This phenomenon may partly be attributed to the findings in this study, that is, the increase in salinity promotes the hydrolysis of organic carbon. In Qinghai Lake, water level monitoring recorded a continuous water level drop of 3.34 m from 1959 to 2005, which caused a rise of 2.05‰ in the average water salinity from 1962 to 2005 (Wang et al., 2008). These records further emphasized the importance of climate change to the sediment C cycle of Qinghai Lake through salinity variation. Collectively, we speculated that sediment C loss would intensify as the lake water becomes concentrated (higher salinity) and phosphorus becomes enriched under climate change and human nutrient pollution, respectively.

Microbial N limitation was predominantly intensified by water TN and decreased with increasing C limitation. Water TN might increase N limitation due to its positive effects on the N cycle, including the activities of NAG and LAP, as inferred from the Pearson correlation analyses and the SEM. The positive effects of water TN on NAG and LAP can be explained as follows: higher TN in water increased the N assimilation of phytoplankton and thus the input of organic nitrogen to sediment in Qinghai Lake (Chen et al., 2012), which led to the release of NAG and LAP by benthic microbes to decompose these organic nitrogen materials. In addition, the decomposition of organic nitrogen (especially proteins) accelerates the formation of an anaerobic status in sediment due to the coupling of nitrification-denitrification caused by ammonium accumulation. This leads to the reduction of ferric iron to ferrous iron and thus the release of soluble reactive phosphorus from iron-bound phosphorus in sediments (Li et al., 2016). Subsequent increases in sediment phosphorus availability changed the microbial nutrient demand from P to N, and hence promoted microbial allocations of EEAs toward NAG and LAP. Furthermore, microbial N limitation was also intensified with the alleviation of C limitation largely because the microbial N demand was stimulated by C addition, which was verified by the positive correlations between sediment TC and LAP (Supplementary Figure 3). Analogously, a review of terrestrial ecosystems indicated that the available soil N becomes increasingly limiting as more carbon is sequestered in plant biomass and soil organic matter in response to rising atmospheric CO_2_ (Luo et al., 2004). It is worth noting that sediment pH exhibited significant or dominant influences on all EEAs except LAP, while it failed to affect microbial metabolic limitations. These results are consistent with previous studies indicating that sediment pH significantly affected EEAs, such as AP activity (Ma et al., 2018), while showed no correlations with enzyme stoichiometry (Luo and Gu, 2018). Comparatively, in soil ecosystems, we now have evidence that soil pH is a strong driver of both soil EEA (Malik et al., 2018; Xu et al., 2020) and enzyme vector length and angle (DeForest and Moorhead, 2020; Wang et al., 2021). The discrepancies in enzymatic stoichiometry caused by alterations in pH between the soil and sediment are probably associated with the reduced sediment pH variation within aquatic sediments caused by the buffering of dissolved carbonates (Sinsabaugh et al., 2009).

Additionally, the bacterial community structure consistently drives the C, N, P, and element cycles and microbial nutrient limitation, while fungal richness only influenced the P cycle. The associations of the microbial community with EEAs and enzyme stoichiometry have been extensively reported in soil ecosystems (Carrara et al., 2018; Dunleavy and Mack, 2021; Wang et al., 2021), while the opposite is true in aquatic sediments. In general, the relative contributions of bacterial and fungal community variations to soil enzyme allocations are dependent on the ecosystem type. For example, in shrubs of the arctic tundra, ectomycorrhizal-associated root enzyme activity profiles are significantly correlated with changes in fungal community composition (Dunleavy and Mack, 2021). In successional subalpine ecosystems after glacier retreat, BG:NAG and BG:AP were tightly linked to the bacterial community composition, while NAG:AP was strongly associated with the fungal community composition (Wang et al., 2021). In a long-term N fertilization experiment in a temperate forest, declines in EEAs following N fertilization were significantly related to a shift in bacterial, but not fungal community composition (Carrara et al., 2018). We proposed that the observed predominant contribution of bacterial rather than fungal community structure to both element cycles and relative investments in enzymes in the sediments of Qinghai Lake may primarily be associated with two factors. (1) Abundance advantage of bacteria, i.e., the number of bacteria in surface sediments is an order of magnitude higher than the number of fungi (Xu et al., 2014). Similar to our results, a sediment study of the mangrove ecosystem revealed that bacteria may be more sensitive to variations in nutrient availability because of the remarkably higher correlation coefficients between bacterial abundance and enzyme stoichiometry than between fungal abundance and enzyme stoichiometry (Luo and Gu, 2018). (2) High activities of bacteria and low resilience of fungi to harsh surroundings (Bapiri et al., 2010; de Vries et al., 2012). For example, a mesocosm experiment in grassland indicated that drought accelerates the destabilizing properties of bacterial (rather than fungal) co-occurrence networks and that bacterial communities are more closely connected to soil functions than fungal communities during recovery from drought (de Vries et al., 2018).

## Conclusion

This study revealed that the benthic microorganisms were colimited by C and N in Qinghai Lake, the largest inland saline lake in China. We showed that the C and P cycles are regulated by sediment pH while the N cycle is affected by water TN and transparency in the sediments of Qinghai Lake. We further found that microbial C limitation was aggravated by increasing salinity and sediment phosphorus, which implied that sediment C loss will intensify as the lake water becomes concentrated and phosphorus becomes enriched under climate change and human-induced nutrient pollution, respectively. For microbial N limitation, water TN had the predominant promoting effect while C limitation had the predominant negative effect. In addition, the bacterial community structure consistently drove the changes in the C, N, and P cycles and in microbial nutrient limitation, while fungi only affected the P cycle through species richness. These results revealed the patterns and drivers of microbial metabolic limitation in saline lakes and further emphasize the potential effects of climate change and human interference on global sediment C storage.

## Data availability statement

The datasets presented in this study can be found in online repositories. The names of the repository/repositories and accession number(s) can be found below: https://www.biosino.org/ : OEP003045.

## Author contributions

WZ: investigation, software, formal analysis, visualization, and writing—original draft. YL, RC, BX, and PX: writing—review and editing. MG: investigation, validation, and formal analysis. JYW: investigation and validation. JJW: supervision, conceptualization, resources, writing—review and editing, data curation, and funding acquisition. All authors contributed to the article and approved the submitted version.

## References

[B1] Anderson N. J. Bennion H. Lotter A. F. (2014). Lake eutrophication and its implications for organic carbon sequestration in Europe. *Glob. Change Biol.* 20 2741–2751. 10.1111/gcb.12584 24677531

[B2] Ao H. Wu C. Xiong X. Jing L. Huang X. Zhang K. (2014). Water and sediment quality in Qinghai Lake. China: a revisit after half a century. *Environ. Monit. Assess.* 186 2121–2133. 10.1007/s10661-013-3522-7 24213639

[B3] Arnosti C. (2011). Microbial extracellular enzymes and the marine carbon cycle. *Annu. Rev. Mar. Sci.* 3 401–425. 10.1146/annurev-marine-120709-142731 21329211

[B4] Bapiri A. Bååth E. Rousk J. (2010). Drying–rewetting cycles affect fungal and bacterial growth differently in an arable soil. *Microb. Ecol.* 60 419–428. 10.1007/s00248-010-9723-5 20635180

[B5] Battin T. J. Besemer K. Bengtsson M. M. Romani A. M. Packmann A. I. (2016). The ecology and biogeochemistry of stream biofilms. *Nat. Rev. Microbiol.* 14 251–263. 10.1038/nrmicro.2016.15 26972916

[B6] Benito-Carnero G. Gartzia-Bengoetxea N. Arias-González A. Rousk J. (2021). Low-quality carbon and lack of nutrients result in a stronger fungal than bacterial home-field advantage during the decomposition of leaf litter. *Funct. Ecol.* 35 1783–1796. 10.1111/1365-2435.13822

[B7] Benstead J. P. Cross W. F. Gulis V. Rosemond A. D. (2021). Combined carbon flows through detritus, microbes, and animals in reference and experimentally enriched stream ecosystems. *Ecology* 102:e03279. 10.1002/ecy.3279 33368179

[B8] Boschker H. Cappenberg T. (1998). Patterns of extracellular enzyme activities in littoral sediments of Lake Gooimeer, The Netherlands. *FEMS Microbiol. Ecol.* 25 79–86. 10.1111/j.1574-6941.1998.tb00461.x

[B9] Carrara J. E. Walter C. A. Hawkins J. S. Peterjohn W. T. Averill C. Brzostek E. R. (2018). Interactions among plants, bacteria, and fungi reduce extracellular enzyme activities under long-term N fertilization. *Glob. Change Biol.* 24 2721–2734. 10.1111/gcb.14081 29488286 PMC5980773

[B10] Chen H. Li D. Zhao J. Zhang W. Xiao K. Wang K. (2018). Nitrogen addition aggravates microbial carbon limitation: Evidence from ecoenzymatic stoichiometry. *Geoderma* 329 61–64. 10.1016/j.geoderma.2018.05.019

[B11] Chen X. Meng X. Song Y. Zhang B. Wan Z. Zhou B. (2021). Spatial patterns of organic and inorganic carbon in Lake Qinghai surficial sediments and carbon burial estimation. *Front. Earth Sci.* 9:714936. 10.3389/feart.2021.714936

[B12] Chen X. Zhu Y. Luo Y. Fu X. (2012). Particular specific features of nitrogen distribution and their effect on alga growth in Qinghai Lake. *J. Saf. Environ.* 12 119–123.

[B13] Cherabier P. Ferriere R. (2022). Eco-evolutionary responses of the microbial loop to surface ocean warming and consequences for primary production. *ISME J.* 16 1130–1139. 10.1038/s41396-021-01166-8 34864820 PMC8940968

[B14] Cui Y. Bing H. Fang L. Jiang M. Shen G. Yu J. (2021). Extracellular enzyme stoichiometry reveals the carbon and phosphorus limitations of microbial metabolisms in the rhizosphere and bulk soils in alpine ecosystems. *Plant Soil* 458 7–20. 10.1007/s11104-019-04159-x

[B15] Curtis P. Adams H. (1995). Dissolved organic matter quantity and quality from freshwater and saltwater lakes in east-central Alberta. *Biogeochemistry* 30 59–76. 10.1007/BF02181040

[B16] de Vries F. T. Griffiths R. I. Bailey M. Craig H. Girlanda M. Gweon H. S. (2018). Soil bacterial networks are less stable under drought than fungal networks. *Nat. Commun.* 9:3033. 10.1038/s41467-018-05516-7 30072764 PMC6072794

[B17] de Vries F. T. Liiri M. E. Bjørnlund L. Bowker M. A. Christensen S. Setälä H. M. (2012). Land use alters the resistance and resilience of soil food webs to drought. *Nat. Clim. Change* 2 276–280. 10.1038/NCLIMATE1368

[B18] DeForest J. L. Moorhead D. L. (2020). Effects of elevated pH and phosphorus fertilizer on soil C, N and P enzyme stoichiometry in an acidic mixed mesophytic deciduous forest. *Soil Biol. Biochem.* 150:107996. 10.1016/j.soilbio.2020.107996

[B19] Dong H. Song Y. Zhang M. (2019). Hydrological trend of Qinghai Lake over the last 60 years: driven by climate variations or human activities? *J. Water Clim. Change* 10 524–534. 10.2166/wcc.2018.033

[B20] Dunleavy H. R. Mack M. C. (2021). Long-term experimental warming and fertilization have opposing effects on ectomycorrhizal root enzyme activity and fungal community composition in Arctic tundra. *Soil Biol. Biochem* 154:108151. 10.1016/j.soilbio.2021.108151

[B21] Fan C. Song C. Li W. Liu K. Cheng J. Fu C. (2021). What drives the rapid water-level recovery of the largest lake (Qinghai Lake) of China over the past half century? *J. Hydrol.* 593:125921. 10.1016/j.jhydrol.2020.125921

[B22] Feng J. Wei K. Chen Z. Lu X. Tian J. Wang C. (2019). Coupling and Decoupling of Soil Carbon and Nutrient Cycles Across an Aridity Gradient in the Drylands of Northern China: Evidence From Ecoenzymatic Stoichiometry. *Glob. Biogeochem. Cycles* 33 559–569. 10.1029/2018gb006112

[B23] Francoeur S. N. Neely R. K. Underwood S. Kuehn K. A. (2020). Temporal and stoichiometric patterns of algal stimulation of litter-associated heterotrophic microbial activity. *Freshwater Biol.* 65 1223–1238. 10.1111/fwb.13442

[B24] Frey S. Six J. Elliott E. (2003). Reciprocal transfer of carbon and nitrogen by decomposer fungi at the soil–litter interface. *Soil Biol. Biochem.* 35 1001–1004. 10.1016/S0038-0717(03)00155-X

[B25] Grace J. B. Schoolmaster D. R. Jr. Guntenspergen G. R. Little A. M. Mitchell B. R. Miller K. M. (2012). Guidelines for a graph-theoretic implementation of structural equation modeling. *Ecosphere* 3:art73. 10.1890/es12-00048.1

[B26] Harbott E. L. Grace M. R. (2005). Extracellular enzyme response to bioavailability of dissolved organic C in streams of varying catchment urbanization. *J. N. Am. Benthol. Soc.* 24 588–601. 10.1899/04-023.1

[B27] Hewins D. B. Chuan X. Bork E. W. Carlyle C. N. (2016). Measuring the effect of freezing on hydrolytic and oxidative extracellular enzyme activities associated with plant litter decomposition. *Pedobiologia* 59 253–256. 10.1016/j.pedobi.2016.09.002

[B28] Hicks L. C. Lajtha K. Rousk J. (2021). Nutrient limitation may induce microbial mining for resources from persistent soil organic matter. *Ecology* 102:e03328. 10.1002/ecy.3328 33705567

[B29] Hill B. H. Elonen C. M. Anderson L. E. Lehrter J. C. (2014). Microbial respiration and ecoenzyme activity in sediments from the Gulf of Mexico hypoxic zone. *Aquat. Microb. Ecol.* 72 107–118. 10.3354/ame01689

[B30] Hill B. H. Elonen C. M. Herlihy A. T. Jicha T. M. Serenbetz G. (2018). Microbial ecoenzyme stoichiometry, nutrient limitation, and organic matter decomposition in wetlands of the conterminous United States. *Wetlands Ecol. Manage.* 26 425–439. 10.1007/s11273-017-9584-5 31073261 PMC6503683

[B31] Hill B. H. Elonen C. M. Jicha T. M. Bolgrien D. W. Moffett M. F. (2009). Sediment microbial enzyme activity as an indicator of nutrient limitation in the great rivers of the Upper Mississippi River basin. *Biogeochemistry* 97 195–209. 10.1007/s10533-009-9366-0

[B32] Hill B. H. Elonen C. M. Jicha T. M. Cotter A. M. Trebitz A. S. Danz N. P. (2006). Sediment microbial enzyme activity as an indicator of nutrient limitation in Great Lakes coastal wetlands. *Freshwater Biol.* 51 1670–1683. 10.1111/j.1365-2427.2006.01606.x

[B33] Hill B. H. Elonen C. M. Seifert L. R. May A. A. Tarquinio E. (2012). Microbial enzyme stoichiometry and nutrient limitation in US streams and rivers. *Ecol. Indic.* 18 540–551. 10.1016/j.ecolind.2012.01.007

[B34] Huang C. Chen Z. Gao Y. Luo Y. Huang T. Zhu A. (2019). Enhanced mineralization of sedimentary organic carbon induced by excess carbon from phytoplankton in a eutrophic plateau lake. *J. Soils Sediments* 19 2613–2623. 10.1007/s11368-019-02261-2

[B35] Huang X. Chen W. Cai Q. (2000). *Survey, Observation and Analysis of Lake Ecology.* Beijing: China Standard Press.

[B36] Kohler T. J. Peter H. Fodelianakis S. Pramateftaki P. Styllas M. Tolosano M. (2020). Patterns and Drivers of Extracellular Enzyme Activity in New Zealand Glacier-Fed Streams. *Front. Microbiol.* 11:591465. 10.3389/fmicb.2020.591465 33329472 PMC7711088

[B37] LaRowe D. E. Arndt S. Bradley J. A. Estes E. R. Hoarfrost A. Lang S. Q. (2020). The fate of organic carbon in marine sediments - New insights from recent data and analysis. *Earth Sci. Rev.* 204:103146. 10.1016/j.earscirev.2020.103146

[B38] Li H. Song C.-L. Cao X.-Y. Zhou Y.-Y. (2016). The phosphorus release pathways and their mechanisms driven by organic carbon and nitrogen in sediments of eutrophic shallow lakes. *Sci. Total Environ.* 572 280–288. 10.1016/j.scitotenv.2016.07.221 27501427

[B39] Liu W. Graham E. B. Dong Y. Zhong L. Zhang J. Qiu C. (2021). Balanced stochastic versus deterministic assembly processes benefit diverse yet uneven ecosystem functions in representative agroecosystems. *Environ. Microbiol.* 23 391–404. 10.1111/1462-2920.15326 33201537

[B40] Luo L. Gu J.-D. (2018). Nutrient limitation status in a subtropical mangrove ecosystem revealed by analysis of enzymatic stoichiometry and microbial abundance for sediment carbon cycling. *Int. Biodeterior. Biodegrad.* 128 3–10. 10.1016/j.ibiod.2016.04.023

[B41] Luo L. Meng H. Gu J. D. (2017). Microbial extracellular enzymes in biogeochemical cycling of ecosystems. *J. Environ. Manage.* 197 539–549. 10.1016/j.jenvman.2017.04.023 28419976

[B42] Luo Y. Su B. Currie W. S. Dukes J. S. Finzi A. C. Hartwig U. (2004). Progressive nitrogen limitation of ecosystem responses to rising atmospheric carbon dioxide. *Bioscience* 54 731–739. 10.1641/0006-35682004054

[B43] Ma S. N. Wang H. J. Wang H. Z. Li Y. Liu M. Liang X. M. (2018). High ammonium loading can increase alkaline phosphatase activity and promote sediment phosphorus release: A two-month mesocosm experiment. *Water Res.* 145 388–397. 10.1016/j.watres.2018.08.043 30173099

[B44] Malik A. A. Puissant J. Buckeridge K. M. Goodall T. Jehmlich N. Chowdhury S. (2018). Land use driven change in soil pH affects microbial carbon cycling processes. *Nat. Commun.* 9:3591. 10.1038/s41467-018-05980-1 30181597 PMC6123395

[B45] Mendonça R. Müller R. A. Clow D. Verpoorter C. Raymond P. Tranvik L. J. (2017). Organic carbon burial in global lakes and reservoirs. *Nat. Commun.* 8:1694. 10.1038/s41467-017-01789-6 29162815 PMC5698497

[B46] Mitri S. Clarke E. Foster K. R. (2016). Resource limitation drives spatial organization in microbial groups. *ISME J.* 10 1471–1482. 10.1038/ismej.2015.208 26613343 PMC5029182

[B47] Moorhead D. L. Rinkes Z. L. Sinsabaugh R. L. Weintraub M. N. (2013). Dynamic relationships between microbial biomass, respiration, inorganic nutrients and enzyme activities: informing enzyme-based decomposition models. *Front. Microbiol.* 4:223. 10.3389/fmicb.2013.00223 23964272 PMC3740267

[B48] Moorhead D. L. Sinsabaugh R. L. Hill B. H. Weintraub M. N. (2016). Vector analysis of ecoenzyme activities reveal constraints on coupled C, N and P dynamics. Soil Biol. *Biochem.* 93 1–7. 10.1016/j.soilbio.2015.10.019

[B49] Nannipieri P. Kandeler E. Ruggiero P. (2002). *Enzyme activities and microbiological and biochemical processes in soil. Enzymes Environ.*

[B50] Orsi W. D. (2018). Ecology and evolution of seafloor and subseafloor microbial communities. *Nat. Rev. Microbiol.* 16 671–683. 10.1038/s41579-018-0046-8 29967420

[B51] Penton C. R. Newman S. (2008). Enzyme-based resource allocated decomposition and landscape heterogeneity in the Florida Everglades. *J. Environ. Qual.* 37 972–976. 10.2134/jeq2007.0248 18453420

[B52] Pritsch K. Raidl S. Marksteiner E. Blaschke H. Agerer R. Schloter M. (2004). A rapid and highly sensitive method for measuring enzyme activities in single mycorrhizal tips using 4-methylumbelliferone-labelled fluorogenic substrates in a microplate system. *J. Microbiol. Methods* 58 233–241. 10.1016/j.mimet.2004.04.001 15234521

[B53] Redfield A. C. (1958). The biological control of chemical factors in the environment. *Am. Sci* 46:230A–221A. 24545739

[B54] Shackle V. Freeman C. Reynolds B. (2000). Carbon supply and the regulation of enzyme activity in constructed wetlands. *Soil Biol. Biochem.* 32 1935–1940. 10.1016/S0038-0717(00)00169-3

[B55] Sinsabaugh R. (2005). “Fungal enzymes at the community scale,” in *The Fungal Community*, eds Dighton J. White J. F. (Boca Raton: CRC Press), 349–360. 10.1201/9781420027891.ch17

[B56] Sinsabaugh R. Moorhead D. (1994). Resource allocation to extracellular enzyme production: a model for nitrogen and phosphorus control of litter decomposition. *Soil Biol. Biochem.* 26 1305–1311. 10.1016/0038-0717(94)90211-9

[B57] Sinsabaugh R. L. Follstad Shah J. J. Hill B. H. Elonen C. M. (2012). Ecoenzymatic stoichiometry of stream sediments with comparison to terrestrial soils. *Biogeochemistry* 111 455–467. 10.1007/s10533-011-9676-x

[B58] Sinsabaugh R. L. Hill B. H. Follstad Shah J. J. (2009). Ecoenzymatic stoichiometry of microbial organic nutrient acquisition in soil and sediment. *Nature* 462 795–798. 10.1038/nature08632 20010687

[B59] Sinsabaugh R. L. Lauber C. L. Weintraub M. N. Ahmed B. Allison S. D. Crenshaw C. (2008). Stoichiometry of soil enzyme activity at global scale. *Ecol. Lett.* 11 1252–1264. 10.1111/j.1461-0248.2008.01245.x 18823393

[B60] Sinsabaugh R. L. Shah J. J. F. (2012). Ecoenzymatic Stoichiometry and Ecological Theory. *Annu. Rev. Ecol. Evol. Syst.* 43 313–343. 10.1146/annurev-ecolsys-071112-124414

[B61] Song K. Wen Z. Xu Y. Yang H. Lyu L. Zhao Y. (2018b). Dissolved carbon in a large variety of lakes across five limnetic regions in China. *J. Hydrol.* 563 143–154. 10.1016/j.jhydrol.2018.05.072

[B62] Song K. Wen Z. Shang Y. Yang H. Lyu L. Liu G. (2018a). Quantification of dissolved organic carbon (DOC) storage in lakes and reservoirs of mainland China. *J. Environ. Manage.* 217 391–402. 10.1016/j.jenvman.2018.03.121 29626842

[B63] Song K. Zhao Y. Wen Z. Fang C. Shang Y. (2017). A systematic examination of the relationships between CDOM and DOC in inland waters in China. *Hydrol. Earth Syst. Sci.* 21 5127–5141. 10.5194/hess-21-5127-2017

[B64] Sparks D. L. (1996). *Methods of Soil Analysis Part 3—Chemical Methods.* Madison, WI: Soil Science Society of America.

[B65] Villalba L. A. Karnatak R. Grossart H.-P. Wollrab S. (2022). Flexible habitat choice of pelagic bacteria increases system stability and energy flow through the microbial loop. *Limnol. Oceanogr.* 67 1402–1415. 10.1002/lno.12091

[B66] Waiser M. J. Robarts R. D. (1995). Microbial nutrient limitation in prairie saline lakes with high sulfate concentration. *Limnol. Oceanogr.* 40 566–574. 10.4319/lo.1995.40.3.0566

[B67] Wang F. Liu J. Yan H. (2008). Analysis on hydrological process of water balance factors in Qinghai Lake. *J. Hydraul. Eng.* 39 1229–1238. 10.13243/jcnki.slxb.2008.11.004

[B68] Wang J. Wu Y. Li J. He Q. Bing H. (2021). Soil enzyme stoichiometry is tightly linked to microbial community composition in successional ecosystems after glacier retreat. *Soil Biol. Biochem.* 162:108429. 10.1016/j.soilbio.2021.108429

[B69] Wang J. Hu A. Meng F. Zhao W. Yang Y. Soininen J. (2022). Embracing mountain microbiome and ecosystem functions under global change. *New Phytol*. 234, 1987–2002. 10.1111/nph.18051 35211983

[B70] Wang X. Cui Y. Zhang X. Ju W. Duan C. Wang Y. (2020). A novel extracellular enzyme stoichiometry method to evaluate soil heavy metal contamination: Evidence derived from microbial metabolic limitation. *Sci. Total Environ.* 738:139709. 10.1016/j.scitotenv.2020.139709 32590116

[B71] Wen Z. Song K. Shang Y. Zhao Y. Fang C. Lyu L. (2018). Differences in the distribution and optical properties of DOM between fresh and saline lakes in a semi-arid area of Northern China. *Aquat. Sci.* 80:22. 10.1007/s00027-018-0572-5

[B72] Wetzel R. G. (2001). *Limnology: lake and river ecosystems.* New York, NY: Elsevier Science.

[B73] Xiao J. Wang S. Y. Zhou Z. J. Zhang Y. Song C. L. Zhou Y. Y. (2018). An enzymatic mechanism for balancing the stoichiometry of nitrogen and phosphorus in a shallow Chinese eutrophic lake. *Sci. Total Environ.* 630 1071–1077. 10.1016/j.scitotenv.2018.02.297 29554728

[B74] Xu W. Pang K.-L. Luo Z.-H. (2014). High fungal diversity and abundance recovered in the deep-sea sediments of the Pacific Ocean. *Microb. Ecol.* 68 688–698. 10.1007/s00248-014-0448-8 25004994

[B75] Xu Z. Zhang T. Wang S. Wang Z. (2020). Soil pH and C/N ratio determines spatial variations in soil microbial communities and enzymatic activities of the agricultural ecosystems in Northeast China: Jilin Province case. *Appl. Soil Ecol.* 155:103629. 10.1016/j.apsoil.2020.103629

[B76] Yang J. Chen Y. She W. Y. Xiao H. Y. Wang Z. Wang H. Y. (2020). Deciphering linkages between microbial communities and priming effects in lake sediments with different salinity. *J. Geophys. Res. Biogeo.* 125:e05611. 10.1029/2019jg005611

[B77] Zhai Z. Luo M. Yang Y. Liu Y. Chen X. Zhang C. (2022). Trade-off between microbial carbon use efficiency and microbial phosphorus limitation under salinization in a tidal wetland. *Catena* 209:105809. 10.1016/j.catena.2021.105809

[B78] Zhang G. Xie H. Kang S. Yi D. Ackley S. F. (2011). Monitoring lake level changes on the Tibetan Plateau using ICESat altimetry data (2003–2009). *Remote Sens. Environ.* 115 1733–1742. 10.1016/j.rse.2011.03.005

[B79] Zhang W. Chen R. Meng F. Yuan H. Geng M. Cheng L. (2021a). Ecosystem functioning is linked to microbial evenness and community composition along depth gradient in a semiarid lake. *Ecol. Indic.* 132:108314. 10.1016/j.ecolind.2021.108314

[B80] Zhang W. Shen J. Wang J. (2021b). Linking pollution to biodiversity and ecosystem multifunctionality across benthic-pelagic habitats of a large eutrophic lake: A whole-ecosystem perspective. *Environ. Pollut.* 285:117501. 10.1016/j.envpol.2021.117501 34380215

[B81] Zhang W. Liu Y. Geng M. Chen R. Wang J. Xue B. (2022). *Data from: The National Omics Data Encyclopedia. (2022).* Available online at: https://www.biosino.org/node/project/detail/OEP003045 (accessed after July 27, 2022).

[B82] Zheng L. Chen H. Wang Y. Mao Q. Zheng M. Su Y. (2020). Responses of soil microbial resource limitation to multiple fertilization strategies. *Soil Tillage Res.* 196:104474. 10.1016/j.still.2019.104474

