# Supplementary material for: Extracellular enzyme stoichiometry reveals carbon and nitrogen limitations closely linked to bacterial communities in China’s largest saline lake
Source: Front Microbiol. 2022 Sep 21;13:1002542. doi: 10.3389/fmicb.2022.1002542 (PMC9532593; doi:10.3389/fmicb.2022.1002542)
Supplement: Supplementary file 1 [file Data_Sheet_1.docx]

Supplementary Information

**The file includes:**

Supplementary Tables (S1-S3)

Supplementary Figure (S1-S6)

**Table S1** Description of environmental variables and extracellular enzyme activities.

|  |  | min | max | median | mean |
| --- | --- | --- | --- | --- | --- |
| Water physiochemical features | Depth (m) | 14.3 | 29.7 | 27 | 25.2 |
|  | Secci depth (SD, m) | 1.8 | 4.3 | 3.5 | 3.4 |
|  | Temperature (ºC) | 13.3 | 15.4 | 14 | 14.1 |
|  | Salinity (‰) | 18.2 | 20.1 | 19.8 | 19.6 |
|  | pH | 7.56 | 8.61 | 8.5 | 8.44 |
|  | Oxidation reduction potential (ORP, mv) | 25.7 | 66.4 | 50.4 | 48.8 |
|  | Dissolved oxygen  (DO, mg L^-1^) | 5.85 | 6.24 | 5.97 | 5.99 |
|  | Turbidity (NTU) | 217 | 468 | 285 | 290 |
|  | Total nitrogen (TN, mg L^-1^) | 0.29 | 2.80 | 0.85 | 1.04 |
|  | Total phosphorus  (TP, mg L^-1^) | 0.01 | 0.09 | 0.03 | 0.04 |
|  | TN:TP | 30.31 | 199.18 | 53.37 | 64.54 |
| Sediment physiochemical features | pH | 9.40 | 10.25 | 9.64 | 9.73 |
|  | Conductivity (µs cm^-1^) | 2690 | 5450 | 3860 | 3917 |
|  | Total carbon (TC, g kg^-1^) | 47.54 | 82.05 | 65.31 | 65.25 |
|  | Total nitrogen  (TN, g kg^-1^) | 1.51 | 4.19 | 2.88 | 2.89 |
|  | Total phosphorus  (TP, g kg^-1^) | 0.50 | 0.73 | 0.63 | 0.62 |
|  | TN:TP | 6.08 | 13.45 | 7.37 | 10.19 |
| Extracellular enzyme activities | β-glucosidase  (BG, nmol MUF·g^-1^·h^-1^) | 82.83 | 1149.47 | 333.79 | 458.12 |
|  | Cellobiohydrolase  (CBH, nmol MUF·g^-1^·h^-1^) | 27.60 | 644.08 | 79.58 | 159.11 |
|  | β-N-acetylglucosaminidase (NAG, nmol MUF·g^-1^·h^-1^) | 47.70 | 258.23 | 94.90 | 114.38 |
|  | Leucine amino peptidase  (LAP, nmol AMC·g^-1^·h^-1^) | 0.00 | 52.52 | 25.13 | 24.90 |
|  | Alkaline phosphatase (AP, nmol MUF·g^-1^·h^-1^) | 28.81 | 230.96 | 91.91 | 92.69 |

**Table S2**. Comparations of the mean and range values of water depth, nutrient contents, extracellular enzyme activity and ecoenzymatic stoichiometry vector features of Qinghai Lake with those in fresh waters including Fuxian Lake in the southwestern Yunnan-Guizhou Pleatau, Hulun Lake in the northern semi-arid area and 38 shallow lakes along the Yangtze-Huaihe River basin of China. Detailed information for the abbreviation of variables is listed in Tables S1. Only means with different letters between lakes are significantly different (*p* ≤ 0.05).

|  | Qinghai Lake  (Sep, 2020) ^†^ | Hulun Lake  (Jun, 2020) ^⁋^ | Fuxian Lake (Jul, 2019) ^‡^ | Lakes along Yangtze-Huaihe River basin (Aug, 2019) ^§^ |
| --- | --- | --- | --- | --- |
| n (site No.) | 23 | 19 | 46 | 80 |
| Water depth (m) | 25.2  (14.3-29.7) ^a^ | 5.7  (3.8-6.4) ^b^ | 68.4  (2.0-155.0) ^c^ | 2.3  (0.3-6.4) ^b^ |
| Water TP (mg L^-1^) | 0.04  (0.01-0.09) ^a^ | 0.13  (0.07-0.17) ^b^ | 0.06  (0.01-0.27) ^a^ | 0.11  (0.02-0.62) ^b^ |
| Water TN (mg L^-1^) | 1.04  (0.29-2.80) ^a^ | 1.12  (0.81-1.35) ^a^ | 0.45  (0.24-1.54) ^b^ | 1.22  (0.28-3.56) ^a^ |
| Water TN:TP | 64.54  (30.31-199.18) ^a^ | 20.42  (16.95-25.46) ^b^ | 23.44  (9.85-54.31) ^b^ | 34.96  (5.82-87.55) ^c^ |
| Sediment TP (g kg^-1^) | 0.62  (0.50-0.73) ^a^ | 0.80  (0.18-1.10) ^a^ | 1.89  (0.50-5.09) ^b^ | 0.84  (0.38-1.87) ^a^ |
| Sediment TC (g kg^-1^) | 65.25  (47.54-82.05) ^a^ | 35.43  (2.32-56.80) ^b^ | 49.35  (9.94-104.07) ^c^ | - |
| Sediment TN (g kg^-1^) | 2.89  (1.51-4.19) ^a^ | 2.06  (0.20-3.40) ^b^ | 3.14  (0.76-5.55) ^a^ | - |
| Sediment TN:TP | 10.19  (6.08-13.45) ^a^ | 4.77  (0.50-11.20) ^b^ | 5.14  (2.19-7.52) ^b^ | - |
| BG | 458.12  (82.83-1149.47) ^a^ | 76.80  (0-260.26) ^b^ | 89.04  (8.92-296.62) ^b^ | 65.46  (0-325.50) ^b^ |
| CBH | 159.11  (27.60-644.08) ^a^ | 7.00  (0-15.80) ^b^ | 2.36  (0.00-30.33) ^b^ | 13.64  (0-122.48) ^b^ |
| NAG | 114.38  (47.70-258.22) ^a^ | 45.50  (0-132.02) ^b^ | 74.03  (5.87-181.45) ^c^ | 39.23  (0-196.86) ^bd^ |
| LAP | 24.90  (0-52.52) ^ab^ | 10.00  (0-172.35) ^a^ | 35.79  (0-373.12) ^b^ | 22.68  (0-251.39) ^ab^ |
| AP | 92.69  (28.82-230.96) ^a^ | 16.90  (5.86-51.37) ^b^ | 87.50  (12.46-164.73) ^a^ | 52.71  (4.47-309.95) ^c^ |
| Ratio of C acquisition enzyme activity (%) | 67  (38-91) ^a^ | 46  (0-93) ^b^ | 31  (7-71) ^c^ | 41  (0-75) ^b^ |
| Ratio of N acquisition enzyme activity (%) | 21  (4-46) ^a^ | 32  (0-73) ^bc^ | 34  (7-72) ^b^ | 29  (0-63) ^c^ |
| Ratio of P acquisition enzyme activity (%) | 13  (3-33) ^a^ | 21  (2-100) ^a^ | 35  (11-80) ^b^ | 30  (3-100) ^bc^ |
| Vector length | 1.125  (0.793-1.348) ^a^ | 0.933  (0-1.366) ^b^ | 0.678  (0.259-1.175) ^c^ | 0.835  (0.496-1.209) ^b^ |
| Vector angle | 42.07  (29.66-48.92) ^a^ | 33.15  (0-56.77) ^b^ | 45.47  (18.94-77.62) ^a^ | 45.06  (20.95-64.46) ^a^ |

^†^This study.

^⁋^19 sites covering the whole Hulun Lake, the largest lake in northern China, were sampled in our previous study (Zhang et al., 2021).

^‡^**^,^** ^§^Unpublished data. There were 1 to 4 sampling sites for each lake along Yangtze-Huaihe River basin.

**Table S3**. Results of multiple stepwise regressions of microbial C and N limitation in relation to abiotic factors.

| Response variable | Model Adjust R^2^ | Model *P* value | Predictor variable | Variable coefficient |
| --- | --- | --- | --- | --- |
| C limitation | 0.498 | 0.002 | Water temperature | 0.37**^*^** |
|  |  |  | Water salinity | 0.32 **^.^** (*P* = 0.06) |
|  |  |  | Sediment TC | -0.38**^*^** |
|  |  |  | Sediment TP | 0.49**^*^** |
| N limitation | 0.402 | 0.009 | Water temperature | -0.26 |
|  |  |  | Water TN | 0.50**^*^** |


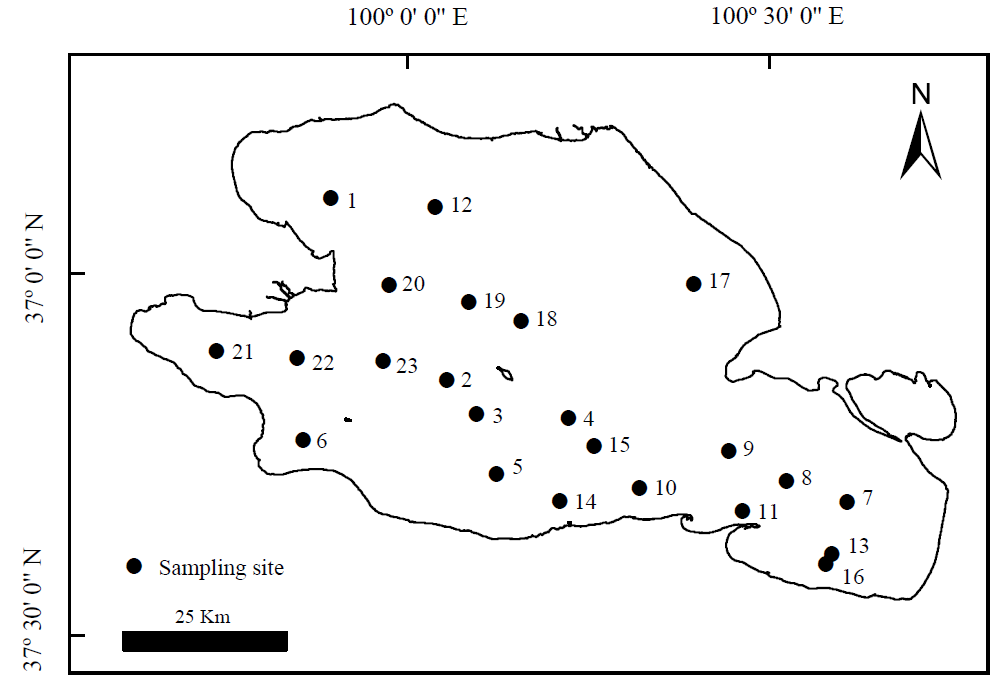


**Figure S1** Sampling map of Qinghai Lake.


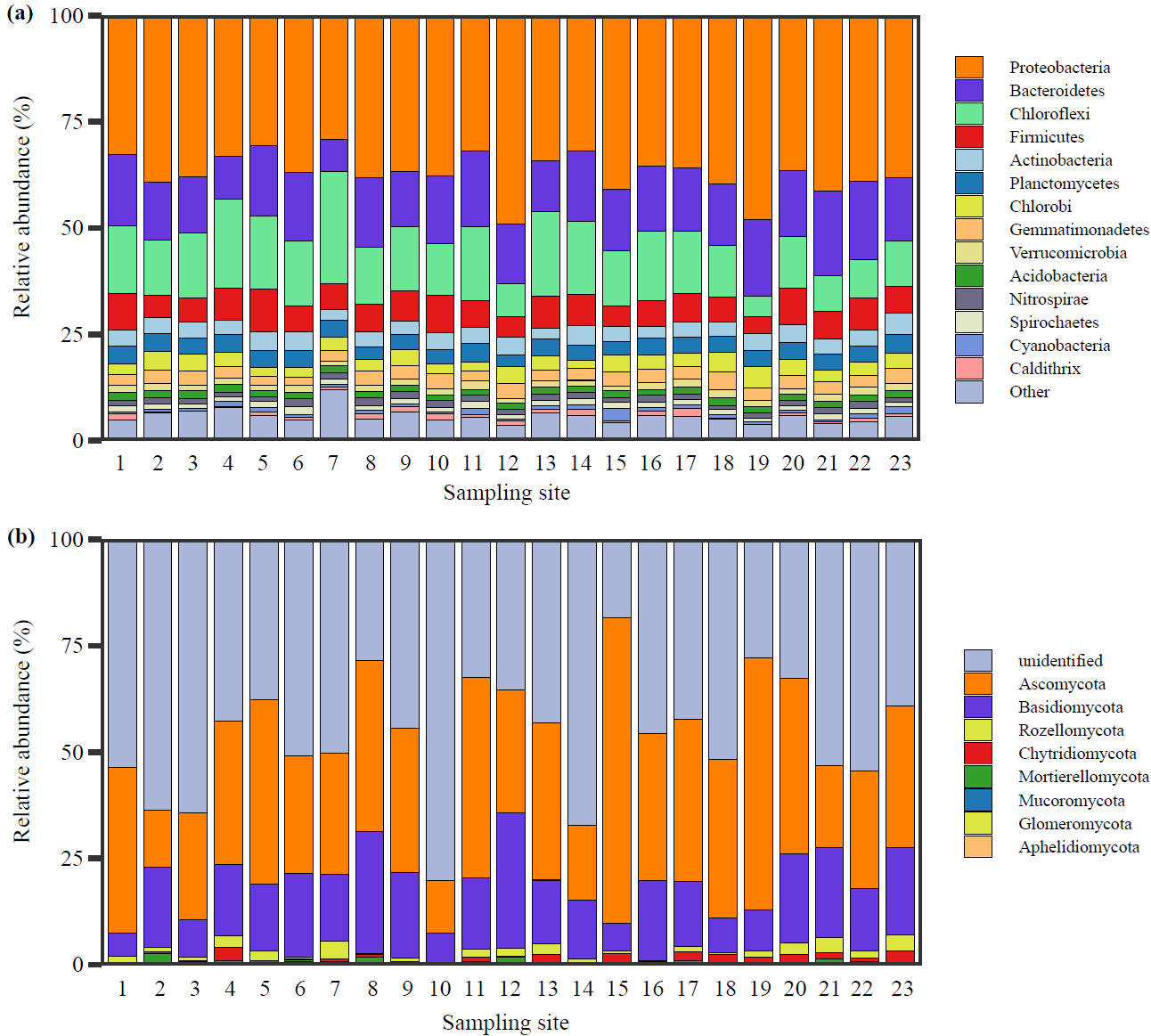
**Figure S2** Stacked bars showing the bacterial (a) and fungal (b) community compositions in phylum level. The x-axis shows each sampling site as shown in the map in Figure S1. “Other” contains phyla with less than 0.5% average of all samples.


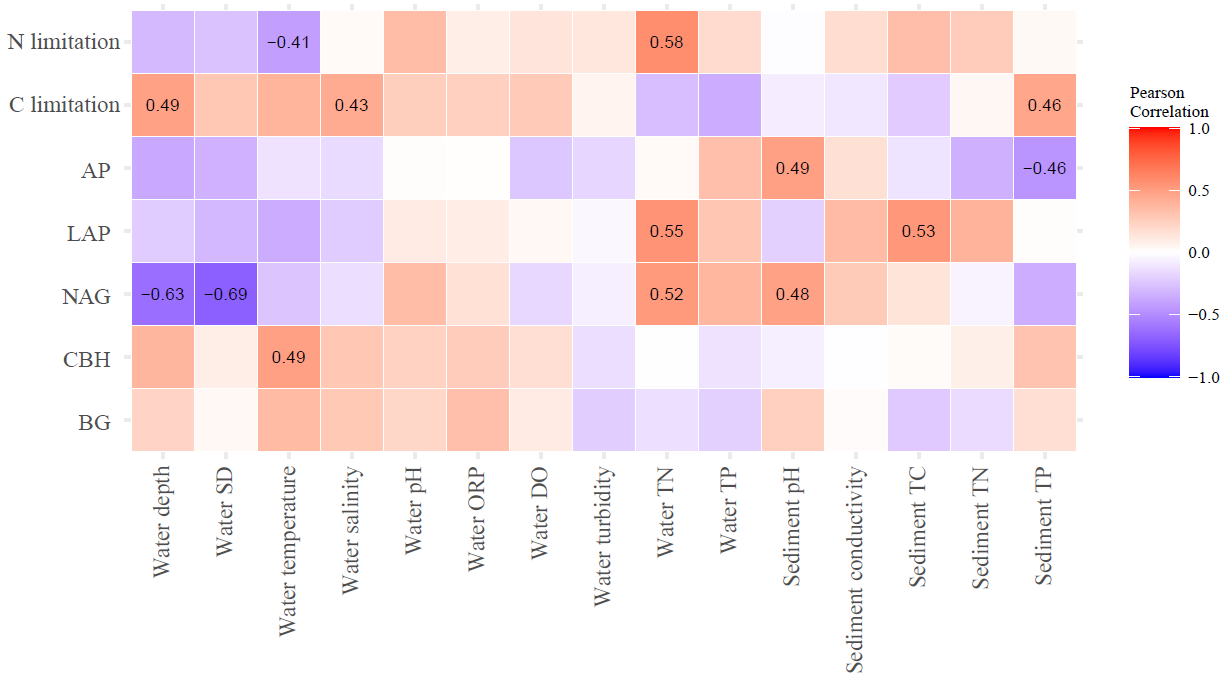
 **Figure S3** Heatmap depicting the correlations of microbial C and N limitations and enzyme activities involved in C (BG and CBH), N (NAG and LAP) and P (AP) cycling with physiochemical factors according to Pearson correlation analyses in Qinghai Lake sediments. BG, β-1,4-glucosidase; CBH, β-D-cellobiosidase; NAG, β-1,4-N-acetylglucosaminidase; LAP, L-leucine aminopeptidase; AP, alkaline phosphatase. Only correlation coefficients with significance level at *p* < 0.05 were shown.


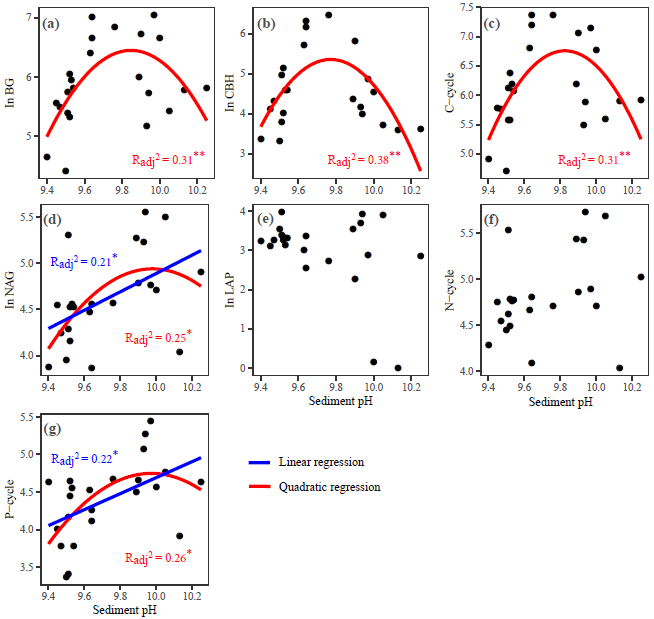


**Figure S4** Linear and quadratic regressions of sediment pH with enzyme activities involved in C-cycle (BG+CBH) (a-c), N-cycle (NAG+LAP) (d-f) and P-cycle (AP) (g). The vertical axes are log-transformed from original data to facilitate the fittings. The blue lines and red curves respectively represent the fitted linear and quadratic regressions, where only significant relationships are shown. The adjusted R^2^ values of the linear and quadratic models are denoted. * *p* < 0.05, ** *p* < 0.01. BG, β-1,4-glucosidase; CBH, β-D-cellobiosidase; NAG, β-1,4-N-acetylglucosaminidase; LAP, L-leucine aminopeptidase; AP, alkaline phosphatase.


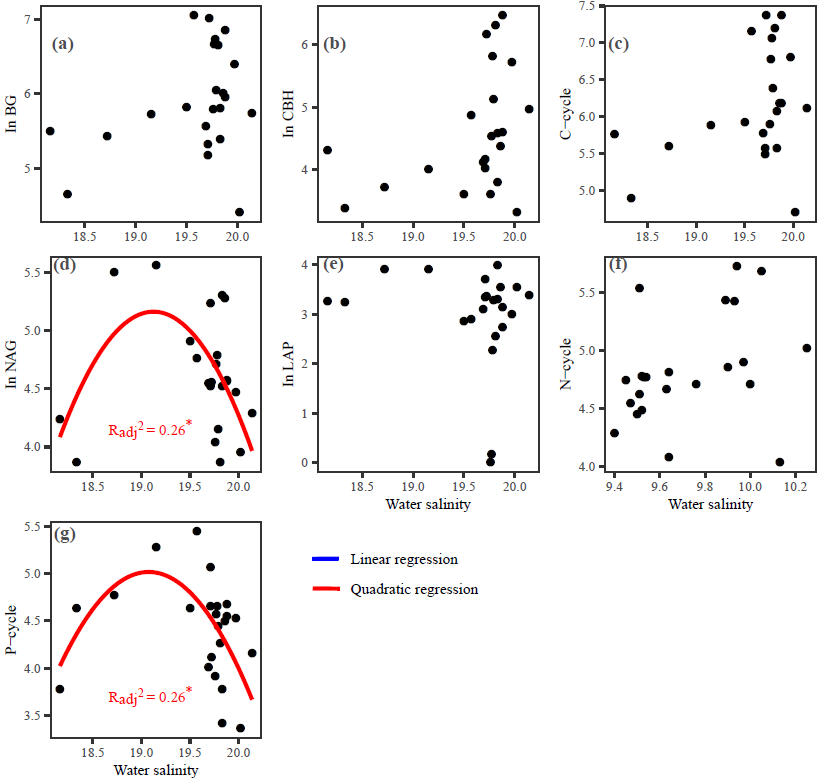


**Figure S5** Linear and quadratic regressions of water salinity with enzyme activities involved in C-cycle (BG+CBH) (a-c), N-cycle (NAG+LAP) (d-f) and P-cycle (AP) (g). The vertical axes are log-transformed from original data to facilitate the fittings. The blue lines and red curves respectively represent the fitted linear and quadratic regressions, where only significant relationships are shown. The adjusted R^2^ values of the linear and quadratic models are denoted. * *p* < 0.05, ** *p* < 0.01. BG, β-1,4-glucosidase; CBH, β-D-cellobiosidase; NAG, β-1,4-N-acetylglucosaminidase; LAP, L-leucine aminopeptidase; AP, alkaline phosphatase.


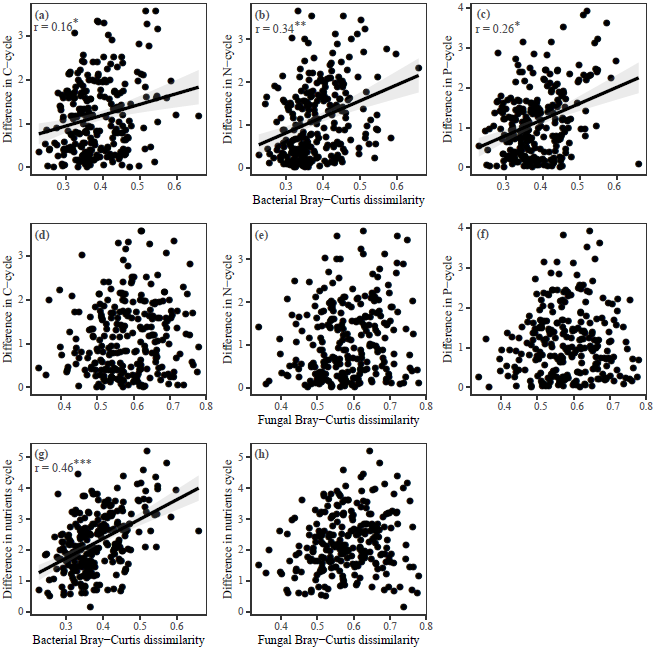


**Figure S6** The relationships between bacterial (a-c) or fungal (d-f) community composition represented by Bray-Curtis dissimilarity and difference in C/N/P-cycle or element-cycle (g-h). The line represents the fitted linear model with 95% confidence intervals indicated by the shaded area, where only significant relationships are shown. The r values of the Mantel tests are denoted. ** p* < 0.05, *** p* < 0.01, **** p* < 0.001.


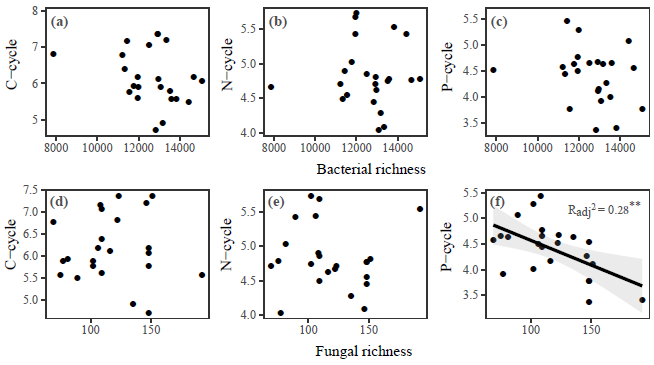


**Figure S7** Linear regressions of bacterial (a-c) and fungal (d-f) richness with C, N or P-cycle. The vertical axes are log-transformed from original data to facilitate the fittings. The line represents the fitted linear regression with 95% confidence intervals indicated by the shaded area, where only significant relationship is shown. The adjusted R^2^ value of the linear model is denoted. *** p* < 0.01.


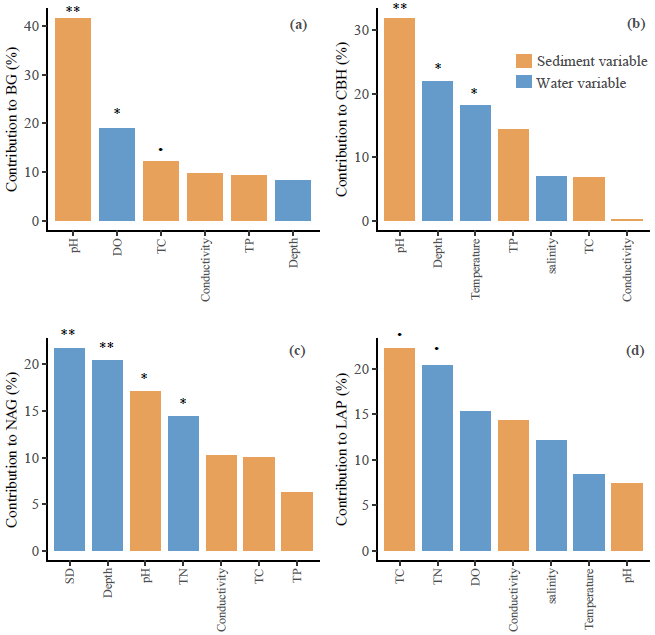


**Figure S8** The relative contributions (%) of abiotic factors on enzyme activities including BG, CBH, NAG and LAP. BG, β-1,4-glucosidase; CBH, β-D-cellobiosidase; NAG, β-1,4-N-acetylglucosaminidase; LAP, L-leucine aminopeptidase. The significance of each variable was shown above the column. ^.^ *p* < 0.1, ** p* < 0.05, *** p* < 0.01.

**References**

Zhang, W., Chen, R., Meng, F., et al., 2021. Ecosystem functioning is linked to microbial evenness and community composition along depth gradient in a semiarid lake. Ecological Indicators. 132, 108314. <https://doi.org/10.1016/j.ecolind.2021.108314>.
